# A Two-Step Bioconversion Process for Canolol Production from Rapeseed Meal Combining an *Aspergillus niger* Feruloyl Esterase and the Fungus *Neolentinus lepideus*

**DOI:** 10.3390/microorganisms5040067

**Published:** 2017-10-14

**Authors:** Elise Odinot, Frédéric Fine, Jean-Claude Sigoillot, David Navarro, Oscar Laguna, Alexandra Bisotto, Corinne Peyronnet, Christian Ginies, Jérôme Lecomte, Craig B. Faulds, Anne Lomascolo

**Affiliations:** 1 INRA Institut National de la Recherche Agronomique, Aix Marseille Univ., UMR1163 BBF Biodiversité et Biotechnologie Fongiques, 163 Avenue de Luminy, 13288 Marseille CEDEX 09, France; elise.odinot@univ-amu.fr (E.O.); jean-claude.sigoillot@univ-amu.fr (J.-C.S.); david.navarro@univ-amu.fr (D.N.); alexandra.bisotto@univ-amu.fr (A.B.); craig.faulds@univ-amu.fr (C.B.F.); 2 Terres Inovia, Parc Industriel, 11 Rue Monge, 33600 Pessac, France; f.fine@terresinovia.fr; 3 Centre International de Ressources Microbiennes, Champignons Filamenteux, CIRM-CF, Case 925, 163 Avenue de Luminy, 13288 Marseille CEDEX 09, France; 4 CIRAD Centre de coopération Internationale en Recherche Agronomique pour le Développement, UMR IATE Montpellier SupAgro-INRA, 2, Place Pierre Viala, 34060 Montpellier, France; oscar.laguna@cirad.fr (O.L.); jerome.lecomte@cirad.fr (J.L.); 5 Terres Univia, 11 rue Monceau, CS60003, 75378 Paris CEDEX 8, France; c.peyronnet@terresunivia.fr; 6 Sécurité et Qualité des Produits d’Origine Végétale, INRA Institut National de la Recherche Agronomique UMR408 SQPOV, Université d’Avignon, 33 rue Louis Pasteur, 84029 Avignon, France; christian.ginies@inra.fr

**Keywords:** *Aspergillus niger*, canolol, feruloyl esterase, *Neolentinus lepideus*, rapeseed meal, sinapic acid

## Abstract

Rapeseed meal is a cheap and abundant raw material, particularly rich in phenolic compounds of biotechnological interest. In this study, we developed a two-step bioconversion process of naturally occurring sinapic acid (4-hydroxy-3,5-dimethoxycinnamic acid) from rapeseed meal into canolol by combining the complementary potentialities of two filamentous fungi, the micromycete *Aspergillus niger* and the basidiomycete *Neolentinus lepideus*. Canolol could display numerous industrial applications because of its high antioxidant, antimutagenic and anticarcinogenic properties. In the first step of the process, the use of the enzyme feruloyl esterase type-A (named AnFaeA) produced with the recombinant strain *A. niger* BRFM451 made it possible to release free sinapic acid from the raw meal by hydrolysing the conjugated forms of sinapic acid in the meal (mainly sinapine and glucopyranosyl sinapate). An amount of 39 nkat AnFaeA per gram of raw meal, at 55 °C and pH 5, led to the recovery of 6.6 to 7.4 mg of free sinapic acid per gram raw meal, which corresponded to a global hydrolysis yield of 68 to 76% and a 100% hydrolysis of sinapine. Then, the XAD2 adsorbent (a styrene and divinylbenzene copolymer resin), used at pH 4, enabled the efficient recovery of the released sinapic acid, and its concentration after elution with ethanol. In the second step, 3-day-old submerged cultures of the strain *N. lepideus* BRFM15 were supplied with the recovered sinapic acid as the substrate of bioconversion into canolol by a non-oxidative decarboxylation pathway. Canolol production reached 1.3 g/L with a molar yield of bioconversion of 80% and a productivity of 100 mg/L day. The same XAD2 resin, when used at pH 7, allowed the recovery and purification of canolol from the culture broth of *N. lepideus*. The two-step process used mild conditions compatible with green chemistry.

## 1. Introduction

Rapeseed is one of the most widely grown oil crops in the world, both in terms of quantities and surface areas, to produce oils for the food or energy (biofuel) industries. Rapeseed meals (RSM), which are the solid by-products of oil extraction processes from seeds, consist mainly of proteins, fibers (pectins, cellulose, hemicelluloses) and minerals [1]. By 2016, the global production of RSM was around 39 million tons [2]. Due to these tonnages, the use and the valorization of these meals became indispensable for a sustainable bio-economy and have been the object of various studies over recent decades. For many years, RSM in the case of digestible varieties have been used mainly as a source of protein hydrolysates for animal feed. In the case of non-digestible cultivars (containing antinutritional factors such as glucosinolates, phytates, polyphenols or erucic acid), RSM were burnt or used as soil fertilizer.

More recently, new alternatives to the traditional use of agri-food by-products for animal feed have emerged, especially for the production of value-added compounds of biotechnological and industrial interest (for a review, see [1]). For instance, meals could be thermo-chemically transformed into natural composite and plastic materials or synthetic additives [3]. Otherwise, RSM proved to be very good physical substrate of microbial growth (porous fibrous structure, adequate chemical composition as source of carbon and nitrogen, minerals and vitamins), as well as being cheap and available in large quantities. In the literature, there are some examples of microbial biotransformations from meals for the production of high value-added molecules, including enzymes, antibiotics, antioxidants, vitamins, lipids, biogas or bioethanol [4,5,6,7,8,9].

**Figure 1:**
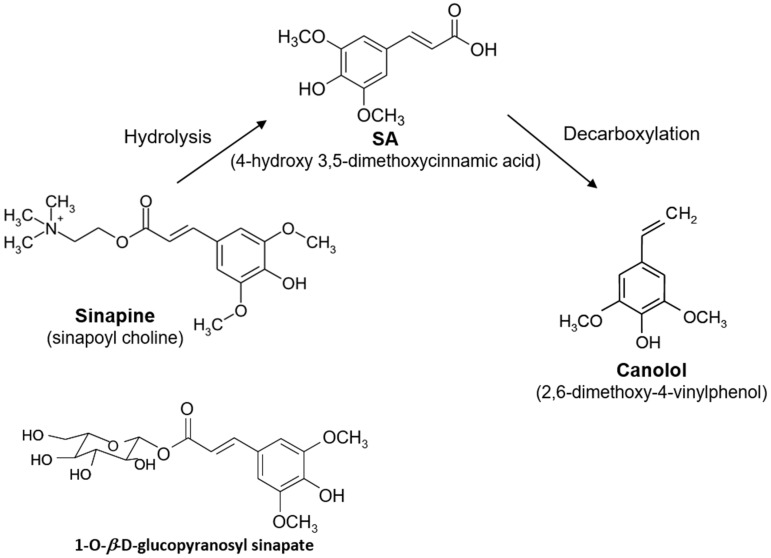
Canolol synthesis from the main esterified forms of SA from rapeseed meal.

Rapeseed meal is particularly rich in phenolic compounds which account for 5 to 18 g/kg [10,11]. The major phenolic compound is sinapic acid (4-hydroxy-3,5-dimethoxycinnamic acid), which is essentially found in esterified forms (Figure 1). The esters of sinapic acid (SA), mainly sinapine (sinapoyl choline) and glucopyranosyl sinapate, represent 90–95% of all the phenolics while the amount of SA in the free form does not exceed 0.03–0.04% [11].

Canolol (2,6-dimethoxy-4-vinylphenol or vinylsyringol) is one of the high value-added molecules that can be obtained from RSM. Discovered and characterized a dozen years ago [12,13], it is the product of SA decarboxylation (Figure 1) which occurrs during the process of oil extraction at high temperature. The canolol content is about 1 mg/g in unrefined crude oils (olive, rapeseed, sesame) but it disappears completely at the end of the refining stages. Canolol is the phenolic compound mostly responsible for the increased oxidative stability of the crude rapeseed oil after the roasting treatment. It has an antioxidant activity comparable to γ-tocopherol, and higher than other natural known antioxidants such as α-tocopherol, vitamin C or β-carotene [12,13]. Moreover, canolol is lipophilic, and shows a better affinity with cell membranes than hydrophilic antioxidants. For instance, canolol was shown to inhibit the intracellular production of free radicals in mammalian cells and to prevent DNA cleavage by these free radicals naturally produced during cell inflammation [14]. Antioxidant and anti-inflammatory effects of canolol have been demonstrated in retinal cell lines [15], and would also play a protective role in cases of inflammation caused by *Helicobacter pylori* gastritis [16]. Canolol specifically reduced the expression level of the gene encoding cyclooxygenase COX-2, a marker of inflammatory activity strongly expressed in tumors where it contributes to inactivate apoptosis. All of these properties seem to confer on canolol a prevention activity against various cancers [17]. In addition, canolol could be the precursor of: (i) natural bio-based monomers (diepoxydized diphenyls) that could advantageously replace the diglycidyl ether of bisphenol A; and (ii) thermoplastic biopolymers [18,19].

It is possible to obtain the decarboxylation of SA into canolol by classical heat treatment (165 °C) of RSM, with yields between 500 and 800 mg/kg, by microwave [20,21,22], or by combining heat, pressure and solvent extraction with alkali [23]. However, the conversion yields remain very low and processes fairly random to scaling-up. Non-oxidative microbial decarboxylation of phenolic compounds to vinyl derivatives has been described historically for ferulic acid (4-hydroxy-3-methoxycinnamic acid) in the residues of fermented products including beer and whisky [24]. This microbial activity involves an intracellular enzyme called phenolic acid decarboxylase (PAD), described in bacilli bacteria and some yeasts till now [25,26,27]. The substrate specificity of the native microbial PADs was described only for ferulic, *p*-coumaric and caffeic acids, in the decreasing order. Recently, a process of SA bioconversion into canolol has been developed with a mutated PAD from *Bacillus pumilus*, in a biphasic (water/toluene) system ensuring continuous extraction and recovery of canolol in the organic phase. The yield was about 3 mg/g meal but SA was obtained by alkaline hydrolysis of the meal followed by two successive extraction steps with hexane, diethyl ether and ethyl acetate [11].

The objective of our work was to develop an alternative strategy based on a two-step bioconversion process to produce up to 1–1.5 g/L canolol from RSM in fungal cultures under mild conditions compatible with green chemistry. In the first step, an *Aspergillus niger* type-A recombinant feruloyl esterase (AnFaeA) enabled the release of free SA from its esterified forms present in the raw meal with a global hydrolysis yield of 68 to 76%. In the second step, 3-day-old submerged cultures of the strain *N. lepideus* BRFM15 were supplied with the recovered sinapic acid as the substrate of bioconversion into canolol by a non-oxidative decarboxylation pathway. Canolol production reached 1.277 g/L with a molar yield of bioconversion of 80% and a productivity of 100 mg/L·day.

## 2. Materials and Methods

### 2.1. Plant Material

Rapeseed meal was provided by the Technical Centre for Oilseed Crops, Grain Legumes and Industrial Hemp (TERRES INOVIA, Pessac, France). It was obtained after oil extraction from rapeseeds at 107 ± 2 °C for 80 ± 5 min.

### 2.2. Chemicals

All the chemicals were purchased from Sigma-Aldrich (Saint-Quentin Fallavier, France), except syringyl alcohol and protocatechuic alcohol (Alfa Aesar, Schiltigheim, France) and sinapine thiocyanate (Wilshire Technologies Inc., Princeton, NJ, USA). Pure canolol was kindly provided by the Laboratory of Agro-polymers and Emerging Technologies of the French National Institute for Agricultural Research (CIRAD-UMR IATE, Montpellier, France). The molecular masses of canolol, SA, sinapine and sinapine thiocyanate were of 180.2, 224.21, 310.37 and 368.45 g/mol, respectively.

### 2.3. Microorganisms and Culture Conditions

The strains *Neolentinus lepideus* BRFM15, *Schizophyllum commune* BRFM823 (deposited by the Université Joseph Fourier, Grenoble, France, as CMPG1552), *Stereum hirsutum* BRFM889, *Aspergillus niger* BRFM281, *A. niger* BRFM451, *A. niger* BRFM766 and *A. niger* BRFM891 were obtained from the CIRM-CF collection (International Centre of Microbial Resources dedicated to Filamentous Fungi, INRA, Marseille, France). The strains were kept on malt agar slants at 4 °C.

For hydroxycinnamic acid bioconversion assays, *N. lepideus* BRFM15, *Schizophyllum commune* BRFM823, and *Stereum hirsutum* BRFM889 were grown in Roux flasks on the following preculture medium: glucose (10 g/L), yeast extract (1 g/L) and bactopeptone (2 g/L). After incubation at 30 °C for 10 days, mycelium from three flasks was collected, mixed with 50 mL sterile water and blended 1 min at 9000 rpm with an Ultra-Turrax T-25 (Janke & Kunkel, GMBM & Co., KG, Staufen, Germany). Mycelial inoculum corresponded to 0.5–0.6 g mycelium dry weight per liter of culture medium. Cultures were grown at 30 °C and 120 rpm in 250-mL Erlenmeyer baffled flasks containing 100 mL culture medium: glucose (20 g/L), yeast extract (0.5 g/L), (NH_4_)_2_C_4_H_4_O_6_ (1.84 g/L), KH_2_PO_4_ (0.2 g/L), CaCl_2_·2H_2_O (0.00132 g/L), MgSO_4_·7H_2_O (0.5 g/L), and thiamine chlorhydrate (0.0025 g/L). After three days of incubation, the hydroxycinnamic acid (either SA, or ferulic, or caffeic, or *p*-coumaric acid) was added as a filter-sterilized solution at a final concentration of 0.3 g/L. Phenolic precursor feeding was performed daily in order to maintain 0.3 g/L as the final concentration in the culture medium.

The strain *A. niger* BRFM281 was grown on the same culture medium, depleted in thiamine chlorhydrate, and with 2.5 g/L maltose instead of 20 g/L glucose. The medium was supplemented with 15 g/L RSM. Cultures were inoculated with 2 × 10^5^ conidiospores per mL. Incubation was carried at 30 °C in 500-mL Erlenmeyer baffled flasks containing 100 mL medium.

The recombinant strains *A. niger* BRFM451, *A. niger* BRFM766 and *A. niger* BRFM891 were, respectively, feruloyl esterase A (AnFaeA), feruloyl esterase B (AnFaeB) and chlorogenate esterase (ChlE) overproducing strains, formerly obtained, in our laboratory, by genetic engineering of the host strain *A. niger* D15#26, as previously described [28,29,30]. In this study, these strains were respectively used to produced batches of AnFaeA, AnFaeB and ChlE, in a culture medium containing: glucose (50 g/L), NaNO_3_ (5.95 g/L), KCl (0.52 g/L), MgSO_4_·7H_2_O (0.49 g/L), trace elements (1000× stock: ZnSO_4_·7H_2_O 21.85 g/L, H_3_BO_3_ 11 g/L, MnCl_2_·4H_2_O 4.95 g/L, FeSO_4_·7H_2_O 5 g/L, CoCl_2_·6H_2_O 1.69 g/L, CuSO_4_·5H_2_O 1.6 g/L, Na_2_MoO_4_·2H_2_O 1.5 g/L, EDTA-Na_2_·2H_2_O 64.76 g/L) in a 0.1 M citrate–sodium phosphate buffer at pH 5 [29].

### 2.4. Accession Numbers of Protein Sequences

The accession numbers of the protein sequences of the fungal PADs predicted from the genome of the strains *N. lepideus* HHB14362, *S. commune* H4-8 and *S. hirsutum* FP-91666 were, respectively, in the NCBI database: KZT30061.1, XP_003032860.1, and XP_007303961.1 [31]. The accession numbers of the protein sequences of AnFaeA, AnFaeB and ChlE were, respectively, in the NCBI database: CAA70510, AJ309807, and ABK62698.

### 2.5. Enzyme Activity Assay

Esterase activity was assayed spectrophotometrically at 37 °C, as previously described [32], by monitoring the A_335_ with respect to the rate of hydrolysis of 0.032 mmol/L of the enzyme substrate in 88 mmol/L of a 3-(*N*-morpholino)propanesulfonic acid (MOPS) buffer (pH 5.5). The substrates used were respectively methyl sinapate and methyl ferulate for AnFaeA, methyl caffeate and methyl *p*-coumarate for AnFaeB, and chlorogenic acid for ChlE. The extinction coefficients at 335 nm were 13,318, 5500, 19,524, 4409, 12,560, 6060, 821, 673, and 15,423 L/(mol·cm) for methyl sinapate, sinapic acid, methyl ferulate, ferulic acid, methyl caffeate, caffeic acid, methyl *p*-coumarate, *p*-coumaric acid, and chlorogenic acid, respectively. Enzyme activity was expressed in nanokatal. One nanokatal of activity is defined as the quantity of enzyme which hydrolyzed 1 nmol of substrate per second. The experiments were performed in triplicate and the standard deviation was lower than 5% of the mean.

### 2.6. Screening of Resins for Aromatic Compound Adsorption

Four resins were used: one polystyrenic resin Amberlite^®^ XAD16, two styrene and divinylbenzene copolymer resins, respectively Sepabeads^®^ SP207 and Amberlite^®^ XAD2, and one styrene divinylbenzene polyaromatic copolymer resin Amberlite^®^ XAD1180N, all purchased from Sigma-Aldrich (Saint-Quentin Fallavier, France). Before use, adsorbents were washed with water and ethanol. The affinity of SA and canolol for these resins was determined in 50-mL solutions containing 5 g wet resin, 178 mg/L SA and 380 mg/L canolol. After an overnight incubation at 30 °C and 120 rpm, the mix was filtered on glass-fibre G1 filters. The resulting filtrate was recovered and then the resin was washed with 50 mL of water and eluted two times with pure ethanol (50 than 20 mL) at 30 °C and 120 rpm for 1.5 h. The aqueous and ethanolic eluates were immediately analyzed by HPLC as described below. The adsorption ratio of each compound for the resin was calculated as follows:

$$adsorption\;ratio\;\left(\%\right)=\frac{\left(C_{0}-C^{*}\right)\times 100}{C_{0}}$$

*C*_0_ initial concentration of sinapic acid or canolol (mmol/L)

C* equilibrium concentration of sinapic acid or canolol (mmol/L)

### 2.7. Enzymatic Hydrolysis of Rapeseed Meal

For the enzymatic hydrolysis assays, batches of recombinant AnFaeA, AnFaeB and ChlE were used. One hundred milliliters of 100 mM MOPS (3-(*N*-morpholino)propanesulfonic acid) buffer (pH 5.5), containing 5 g RSM was incubated for 1 h at 30 °C and 150 rpm for homogenization and then either 30.5 nkat AnFaeA or 18.2 nkat ChlE or 26.1 nkat AnFaeB was added. The enzymatic hydrolysis assay was then run at 37 °C and 150 rpm for 1 to 4 h. The reference reaction mixture was performed in the same conditions but in the absence of any enzyme. The release of sinapine and SA from RSM was followed by HPLC analysis of the buffer mixture, according to the method described below.

### 2.8. Batch Production of Sinapic Acid from Rapeseed Meal

Four hundred and ten grams of RSM (dry weight) were incubated with 17,640 nkat AnFaeA (i.e., 39 nkat AnFaeA per gram of meal) and 8 L of 100 mM MOPS buffer (pH 5), with an agitation of 500 rpm in a 10-L Pierre Guérin Tryton bioreactor of standard geometry (Pierre Guérin technologies, Mauze, France), for 3.5 h at 55 °C. The mechanically agitated tank was equipped with a pitch blade impeller (6.5 cm in diameter) and a VMI centripetal impeller (5.5 cm in diameter, VMI Rayneri, Montaigu, France) separated each other by 11.8 cm. The residual meal was then removed by filtration and the pH of the resulting supernatant was adjusted to pH 4 with HCl before an overnight incubation with 500 g resin XAD2, at 30 °C and 160 rpm, in order to specifically adsorb SA. The elution of SA from the resin was performed with 2 L ethanol. Ethanol was further evaporated under vacuum at 30 °C to obtain a concentrated SA solution of 25 g/L.

### 2.9. Characterization of Rapeseed Meal

Dry matter content of rapeseed meal (RSM) was determined by drying at 110 °C until constant mass was obtained.

Lipid content of RSM was determined by hexane extraction using a ratio of 10% (*w/v*) meal in solvent. Extraction was carried out by stirring the substrate in the solvent for 3 days at room temperature. After filtration on a G2 fritted glass filter, hexane was evaporated to dryness and the lipid residue was weighed.

For the determination of total phenolic content of meal, 0.5 g RSM and 50 mL of 70% (*v/v*) aqueous ethanol were incubated at 75 °C for 1 h under continuous stirring. The clear phenolic extract was collected after centrifugation (4000 rpm, 20 min, Fiberlite^®^ F15S-6x100y rotor, Sorvall ST40 centrifuge, Thermoscientific, Illkirch, France) to discard proteins. The total phenolic content was determined colorimetrically at 750 nm by the Folin–Ciocalteu reagent, using SA as calibration standard. Results of analyses were expressed as mg/L or mg/g dry matter of SA equivalents (SAE). The calibration curve was established from 0 to 100 mg/L.

The total amount of sinapine and free SA present in the raw meal was determined as follows: 50 mg RSM was added to 5 mL methanol and incubated at 75 °C for 20 min under stirring. After centrifugation (3000× *g*, 5 min), the organic phase was analyzed by HPLC to calculate the amount of sinapine and free SA. The total amount of SA, initially present in the raw RSM as various esterified forms and potentially releasable, was determined after alkaline hydrolysis. Fifty milligrams of RSM were diluted with 1.5 mL of methanol. Then, 6 mL of NaOH 2N were added to the mixture for incubation at 30 °C for 30 min. At the end of the incubation, the mixture was acidified at pH 2 using 4N HCl and 300 mg NaCl. Samples were extracted three times with ethyl acetate (2 mL) by vortexing for 2 min. After each extraction, the samples were centrifuged (3000× *g*, 5 min) and the supernatants collected. The organic phases were pooled and dried under nitrogen flow. The residue was dissolved in a final volume of 0.5 mL methanol/water (2:1, *v/v*) prior to HPLC analysis for total SA quantification. HPLC analysis was performed at 30 °C on a model XR UFL Shimadzu LC-20AD equipped with a SPD-M20A variable UV detector (Kyoto, Japan). Separations were achieved on a C18 reversed-phase column (C18 121-2546 5 µm, 4.6 × 250 mm, Phénomenex, Le Pecq, France). The flow rate was 1 mL/min. The used mobile phases were solvent A: water acidified by 0.1% acetic acid (*v/v*), and solvent B: methanol acidified by 0.1% acetic acid (*v/v*). The gradient changed as follows: solvent B started at 15% for 5 min, increased to 80% in 25 min, then to 100% in 1 min until the end of running (35 min). The quantification of compounds was performed by external standard calibration at 323 and 328 nm for SA and sinapine, respectively, using commercial SA and sinapine thiocyanate.

### 2.10. Extraction, Derivatization and Gas Chromatography–Mass Spectrometry (GC–MS) of the Monomeric Phenolics from N. lepideus Culture Broth

Clarified supernatants of *N. lepideus* BRFM15 cultures were adjusted to pH 3 with HCl and twice extracted with ethyl acetate (1:1, *v/v*). The pooled ethyl acetate phase was evaporated to dryness and the residue dissolved in 2 mL of ethyl acetate for subsequent analyses. One mL was then completely dried and submitted to the following procedure for derivatization prior to GC-MS analysis: 297 µL of *N*-methyl-*N*-(trimethylsilyl)fluoroacetamide (MSTFA) and 3 µL of 2% (*w/v*) methoxyamine hydrochloride in pyridine were incubated with the sample for 15 min at 60 °C. Trimethylsilyl derivatives of monomeric phenolic extracts were then analyzed by GC–MS using an Agilent 6890N GC-5973N mass detector (Agilent Technologies, Massy, France). The column was an Agilent DB5-MS (30 m × 0.25 mm i.d., 0.25 µm film thickness) with an inlet system using the split 1:20 injection technique. Injector temperature was 250 °C. Helium was used as the column carrier gas at a constant flow rate of 36 cm/s. The oven temperature was held at 70 °C for 2 min, then raised to 280 °C at a rate of 10 °C/min and held at 280 °C for 5 min, then raised to 300 °C at a rate of 10 °C/min and held at 300 °C for 5 min. The electron impact energy was set at 70 eV; the ion source and quadrupole temperatures were 230 and 150 °C, respectively. EI mass spectra ranged from 40 to 650 amu. The identification of compounds was carried out by comparing the mass spectra with those of the NIST library. The commercial standards of the targeted aromatic compounds (except vinylcatechol which was unavailable) were also analyzed using the same method.

### 2.11. High Performance Liquid Chromatography (HPLC) Analysis of the Monomeric Phenolics from Fungal Culture Broth

Daily HPLC analysis of monophenolics from the culture medium of the studied fungal species was performed at 220 nm and 30 °C on a model Agilent1100 (Agilent Technologies, Massy, France) equipped with a variable UV/Vis detector and a 100-position autosampler autoinjector. Separations were achieved on a C30 reversed-phase column (YMC™ Carotenoid 3 µm, 4.6 × 150 mm, Waters, Guyancourt, France). The flow rate was 0.8 mL/min. The used mobile phases were solvent A: water acidified by 0.05% phosphoric acid and acetonitrile (95:5, *v/v*), and solvent B: acetonitrile 100%. The gradient changed as follows: solvent B started at 10% for 4 min, increased to 40% in 9 min, then to 100% in 1 min until the end of running (18 min). The Agilent1100 ChemStation processed the data, and the quantification was performed by external standard calibrations.

All experiments were run in duplicate or triplicate and the standard deviation of the analyses was less than 5%.

## 3. Results

### 3.1. Characterization of RSM

The moisture, fat and total phenolic contents of the RSM used in this study were of 10.0 ± 0.1% dry matter (DM), 1.2 ± 0.06% DM, and 19 ± 1.3 mg SAE/g DM, respectively. These data were consistent with the values available in the literature (for a review, see [1]). The sinapine and free SA content was determined after a methanolic extraction of the RSM phenolics and an alkaline hydrolysis, and was of 8 ± 0.53 mg/g DM (25.8 ± 1.7 µmol/g DM) and 0.49 ± 0.01 mg/g DM (2.2 ± 0.04 µmol/g DM) respectively. After alkaline hydrolysis, all the esterified forms of SA were hydrolyzed into a total of 9.77 ± 0.27 mg free SA/g DM (43.6 ± 1.2 µmol free SA/g DM). The latter value represented the total amount of the potentially releasable SA per gram of meal. It was used as the 100% reference for the calculations of enzymatic hydrolysis yields.

### 3.2. Release of Free SA from Meal by A. niger Esterases

When grown in the presence of appropriate natural inducers, the strain *A. niger* BRFM281 has already been shown to produce feruloyl esterases able to release free hydroxycinnamic acids, including ferulic, *p*-coumaric and caffeic acids, from plant complex biomass such as cereal bran and sugar beet pulp [33]. In our study, the strain *A. niger* BRFM281 was grown in the presence of RSM as carbon source and enzyme inducer in the culture medium. AnFaeA, AnFaeB, and ChlE activities could be detected from day 3 to day 11 of cultivation on RSM, with maximal production of 0.67 nkat/mL AnFaeA, 0.44 nkat/mL AnFaeB and 0.29 nkat/mL ChlE on day 10, and SA was effectively released in the culture supernatant. However, SA was immediately converted into syringic acid (4-hydroxy-3,5-dimethoxybenzoic acid), certainly via a β-oxidation-type pathway as previously described for the conversion of ferulic acid into vanillic acid [34]. It is also noteworthy that no canolol could be detected in the culture medium of *A. niger*. Consequently, the release of free SA from RSM was further carried out by using the recombinant enzymes AnFaeA, AnFaeB and ChlE, formerly well-characterized in our laboratory [28,29,30], instead of the fungus itself.

**Figure 2:**
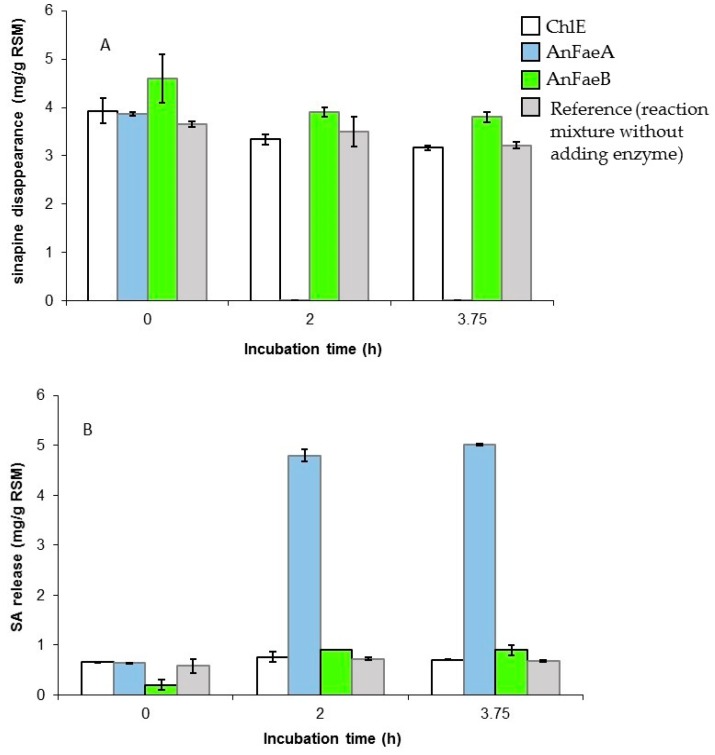
Time course of sinapine disappearance (**A**) and SA increase (**B**) in the reaction mixture of RSM hydrolysis as a function of the cinnamoyl esterase tested.

**Figure 3:**
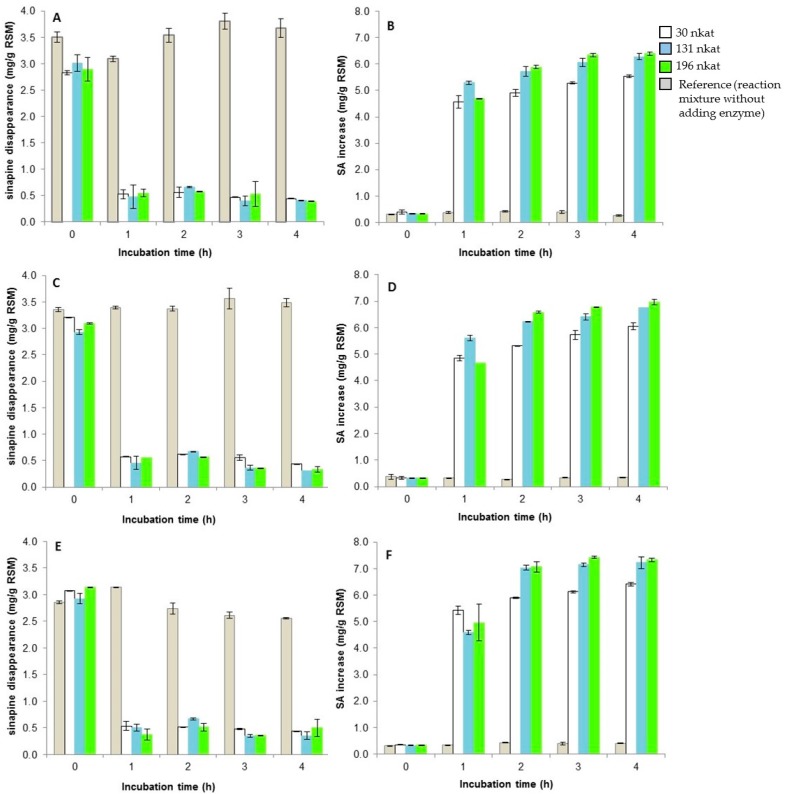
Time course of sinapine disappearance and SA increase in the reaction mixture of hydrolysis as a function of the amount of AnFaeA and temperature. (**A**,**B**): 37 °C, (**C**,**D**): 45 °C, (**E**,**F**): 55 °C.

The enzymatic hydrolysis assays were first performed by mixing 5 g RSM with either 30.5 nkat AnFaeA or 18 nkat ChlE or 26.1 nkat AnFaeB at 37 °C. These activities corresponded, respectively, to total AnFaeA, ChlE and AnFaeB activities previously quantified in 100 mL of RSM-induced *A. niger* BRFM281 cultures, on day 4 of cultivation before any conversion of SA into syringic acid. The results of RSM hydrolysis are shown on Figure 2. It is noteworthy that RSM had been homogenized for one hour in the reaction buffer before adding the enzyme. This preliminary incubation solubilized about 50% of the amount of sinapine and 100% of the free SA initially present in the meal. AnFaeB and ChlE did not enable the release of free SA from RSM while, with AnFaeA, a total disappearance of sinapine (100% hydrolysis) was observed after 2 h of incubation, correlated with a significant increase of SA in the reaction medium up to 5 mg/g DM (Figure 2). In this case, the global hydrolysis yield was about 50% (compared to the value of 9.77 mg/g DM). Then, AnFaeA was chosen for further optimization by varying the enzyme/meal ratio as well as the reaction temperature: 30.5, 131 or 196 nkat of AnFaeA was used at 37, 45 or 55 °C (Figure 3).

After 2 h of incubation, the results showed that, for a given amount of enzyme, the higher temperature of the reaction, the greater quantity of SA released, with an increase from 4.8 to 6.0 mg/g DM with 30.5 nkat AnFaeA, 5.7 to 7 mg/g DM with 131 nkat AnFaeA, and 5.9 to 7.1 mg/g DM with 196 nkat AnFaeA. For a given temperature, the higher quantity of enzyme, the greater SA released, with an increase from 4.8 to 5.8 mg/g DM at 37 °C, 5.2 to 6.5 mg/g DM at 45 °C, and 5.9 to 7.1 mg/g DM at 55 °C. These results were confirmed after 3 and 4 h of incubation. The highest amount of free SA released (7.4 mg/g DM) was obtained at 55 °C in the presence of 196 nkat AnFaeA after 3–4 h of incubation (Figure 3). Under these conditions, the hydrolysis yield was about 76% (compared to the value of 9.77 mg/g DM). Some other compounds, such as kaempherol derivatives, and esters of SA including di- and trisinapoyl esters were detected in the enzymatic incubation mixture (Figure S1). AnFaeA hydrolyzed not only sinapine but also glucopyranosyl sinapate, di- and trisinapoyl gentiobioside, and disinapoyl *β*-glucopyranoside while it did not exhibit any activity on kaempherol derivatives. The total SA, present as the free and the choline ester forms represented 28 µmol/g DM in the raw RSM (Table S1). After 4 h of AnFaeA hydrolysis, the total SA (the free form only) amount was of 29.88 µmol/g DM (Table S1). The molar mass balance thus showed that all the initial sinapine present in the raw RSM was enzymatically hydrolyzed in addition to some other SA esters (Figure S1) counting for about 2 µmol EAS/g DM. Moreover, the analysis of the residual enzymatically-treated RSM (after 4h of AnFaeA treatment) confirmed that there was no sinapine at all in the residual meal.

### 3.3. Screening of Fungal Strains able to Biotransform Hydroxycinnamic Acids into the Corresponding Vinyl Compounds

**Figure 4:**
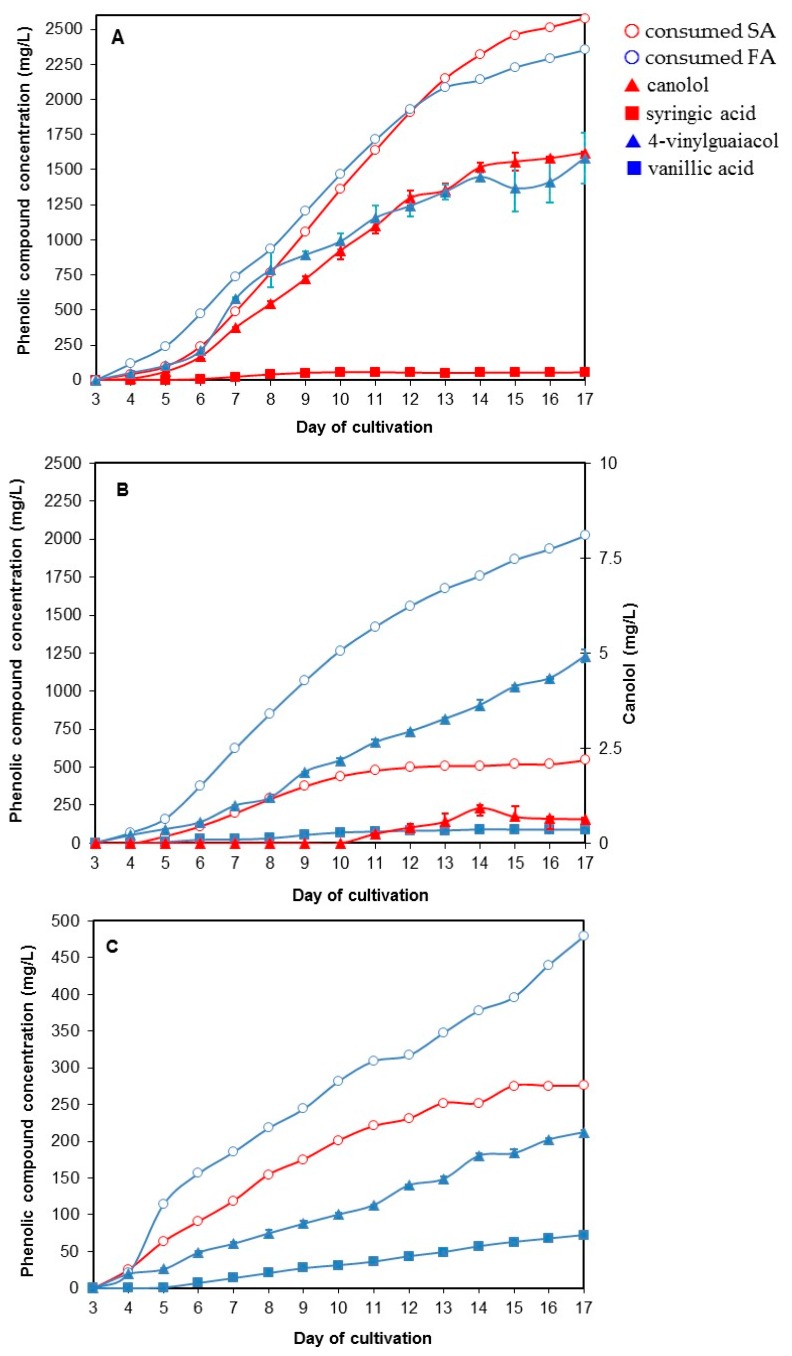
SA and FA metabolism by *N. lepideus* BRFM 15 (**A**), *S. commune* BRFM823 (**B**) and *S. hirsutum* BRFM889 (**C**): consumption of SA or FA in relation to the production of canolol and syringic acid, and VG and vanillic acid, respectively.

Among the currently available and public sequenced genomes of Higher Fungi [31], few contain putative annotated PAD, including some species of *Aspergillus* and *Penicillium* for the Ascomycota phylum, and the species *N. lepideus*, *S. commune*, and *S. hirsutum* for the Basidiomycota phylum. The protein sequences of the fungal PADs predicted from the strains *N. lepideus* HHB14362, *S. commune* H4-8 and *S. hirsutum* FP-91666 from genomes [31] showed no more than 45% similarity to known sequences of bacterial or yeast PADs. In addition, the predicted protein sequence of the *N. lepideus* HHB14362 PAD showed no more than 40% similarity with those of *S. commune* and *S. hirsutum* (Figure S2). The strains *N. lepideus* BRFM15, *S. commune* BRFM823 and *S. hirsutum* BRFM889, available in the CIRM-CF collection, were thus chosen for testing their ability to biotransform either commercial SA or ferulic acid (4-hydroxy-3-methoxycinnamic acid) into canolol and 4-vinylguaiacol (2-methoxy-4-vinylphenol) respectively. Each acid was daily added from day 3 to the end of cultivation in order to maintain a final concentration of 300 mg/L in the culture medium. Sequential addition of the bioconversion precursor was performed because of the relative toxicity of phenolic compounds towards fungal growth in a general way. The results are shown on Figure 4A–C. Only the strain *N. lepideus* BRFM15 was able to efficiently biotransform both SA and ferulic acid (FA) into up to 1.5 g/L canolol and 4-vinylguaiacol (VG), respectively. The identification of canolol and VG was confirmed by GC–MS analysis (Figures S3–S6) The production of canolol from SA by *N. lepideus* BRFM15 started from day 4 and increased up to 1.5 g/L on day 14 (productivity 108 mg/L day) with a molar yield of 81%. About 50 mg/L syringic acid (4-hydroxy-3,5-dimethoxybenzoic acid) was concomitantly synthesized. In this case, the specific growth rate µ of *N. lepideus* BRFM15 was 0.57 day^−1^. *S. commune* and *S. hirsutum* slowly consumed SA compared to *N. lepideus*. Traces of canolol (lower than 1 mg/L) could be detected in the culture medium of *S. commune* grown in the presence of SA whereas *S. hirsutum* did not produce any canolol. However, both strains biotransformed FA into VG. The production of VG started from day 4 of cultivation and reached 1.45 g/L on day 14 (productivity 103 mg/L day) with a molar yield of 87% for *N. lepideus* BRFM15, 1.03 g/L on day 15 (productivity 69 mg/L day) with a molar yield of 71% for *S. commune* BRFM823, and 69 mg/L on day 16 (productivity of 13 mg/L day) with a molar yield of 60% for *S. hirsutum* BRFM889. The quantity and yield of VG obtained with *S. commune* and *S. hirsutum* remained thus lower than those obtained with *N. lepideus*. The bioconversion of FA by *S. commune* and *S. hirsutum* led also to the synthesis of vanillic acid (4-hydroxy-3-methoxybenzoic acid) in amounts lower than 90 mg/L. The strain *N. lepideus* BRFM15 was selected for subsequent studies of SA bioconversion into canolol.

### 3.4. Selection of a Specific Adsorbent for SA or Canolol Recovery

The use of a specific adsorbent should independently permit: (i) the recovery and concentration of SA, released from RSM after AnFaeA hydrolysis and further used as bioconversion precursor for feeding *N. lepideus* cultures; (ii) the recovery of canolol from the culture broth of *N. lepideus* BRFM 15 after bioconversion of SA.

In the first case, the choice of the most suitable adsorbent was thus determined by assessing the ability to adsorb a maximum of SA. Since the pK_A1_ and pK_A2_ of SA were 4.6 and 9.95 respectively, the hydrophobic resins were tested at pH 4 where SA was totally in the protonated form. In the second case, the selected adsorbent should trap high amounts of canolol and show the lower affinity for SA. The adsorption ratios of four resins, XAD1180, XAD16, XAD2, and SP207, for SA or canolol, are shown in Table 1. At pH 4, all the resins showed high affinity for SA. At pH 7, the most selective adsorbent was XAD2 with which 97% of the total canolol was recovered against only about 31% of SA. The XAD2 resin thus showed the best compromise for the use in both steps of the process.

A subsequent assay of canolol batch production and recovery was performed with a 4.5-L culture of *N. lepideus* BRFM15 supplemented with commercial SA. After 13 days of cultivation where the concentration of SA was maintained daily at 300 mg/L in the medium, the supernatant (4.2 L) contained 1.74 g residual SA and 4.54 g canolol. After separation from the mycelium, it was then adjusted to pH 7, added to 420 g XAD2 resin, and incubated overnight at 30 °C and 120 rpm. Then, the compounds were eluted from XAD2 with methanol. HPLC analysis of the resulting methanolic solution showed that it contained only canolol and SA, with quantities of 3.7 g canolol (82.5%) and 0.787 g SA (17.5%).

### 3.5. The Two-Step Bioconversion Process: Batch Production of Natural SA from Rapeseed Meal and Bioconversion into Canolol

In a first step, 410 g (dry matter) of RSM was incubated with 17,640 nkat of AnFaeA (i.e., 39 nkat AnFaeA per gram of meal) in 8 L of 100 mM MOPS buffer (pH 5), at 55 °C for 3.5 h with an agitation of 500 rpm. At the end of the incubation period, 2.71 g SA was released which corresponded to a total hydrolysis of sinapine and a global hydrolysis yield of 68%. After separation from the residual meal, the enzymatic hydrolysis supernatant, which pH was adjusted to 4, was then incubated overnight at 30 °C with 500 g XAD2 resin with orbital stirring of 160 rpm. The elution of bound SA was carried out with ethanol, and resulted in the recovery of 2.46 g SA, which corresponded to 91% of the amount of the SA released from the raw meal. Ethanol was then evaporated under low pressure at 30 °C, in order to obtain an ethanolic solution of natural SA with a concentration of 25 g/L.

**Figure 5:**
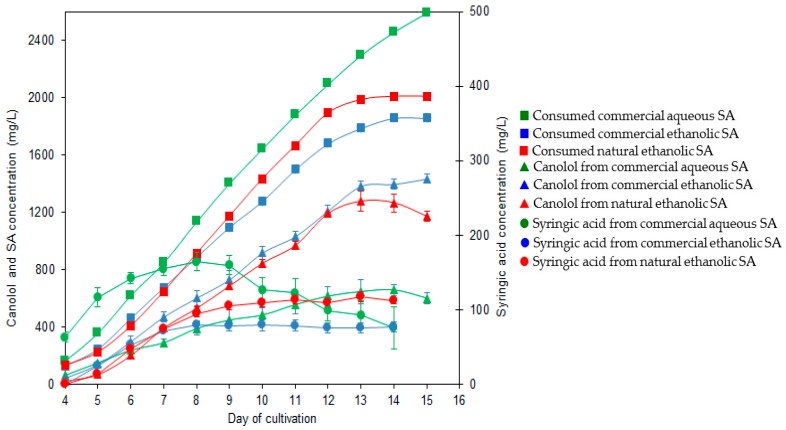
Comparison of SA metabolism by *N. lepideus* BRFM15 supplemented with different sources of SA. Cultivations were carried out in the presence of aqueous commercial SA (green symbols), ethanolic commercial SA (blue symbols), and natural ethanolic SA from RSM (red symbols). Consumption of SA in relation to the production of canolol and syringic acid.

In a second step, the natural SA ethanolic solution was subsequently added to 3-day-old cultures of *N. lepideus* BRFM15 and then fed daily to maintain a final concentration of 300–400 mg/L in the culture medium. The results were compared to the equivalent bioconversion process performed with commercial SA added as either an ethanolic or an aqueous solution (Figure 5). The production of canolol from natural SA started on day 4 of cultivation to reach the maximum concentration of 1.277 g/L on day 13 with a productivity of 100 mg/L day and a molar yield of 80%. It is noteworthy that the supply of either natural or commercial SA as an ethanolic solution made it possible the decrease of syringic acid synthesis by a factor of 1.6–2, during the first 9 days of cultivation, compared with commercial SA dissolved in water.

## 4. Discussion

RSM is a cheap (250–350 €/ton in Europe) and abundant raw material. Europe is the world’s largest producer of RSM ahead of China, Canada and India [2]. Given that the annual European production of RSM is about 13 million tons a year and that RSM content in SA is about 1% DM, there are about 130,000 tons of SA annually available. Canolol is a derivative of SA with numerous applications in pharmaceutical, cosmetic and food industries because of its high antioxidant, anti-mutagenic and anticarcinogenic properties. In this work, a two-step bioconversion process of natural SA from RSM to canolol was elaborated combining the complementary potentialities of two filamentous fungi, the micromycete *A. niger* and the basidiomycete *N. lepideus*. In the first step, the AnFaeA recombinant enzyme from *A. niger* permitted the hydrolysis of the esterified forms of SA in meal (mainly sinapine and and glucopyranosyl sinapate) and its release in the free form. In the second step, free SA was non-oxidatively decarboxylated into canolol by *N. lepideus*.

Obtaining free SA from RSM can be carried out by alkaline hydrolysis and methanol extraction [22,35], but in harsh conditions (high temperature, strong base and toxic solvent), which could be incompatible with a green process. The use of commercial esterase preparations for SA extraction from meal has already been carried out but for analytical purposes only till now [35]. In our work, we exploited the ability of the species *A. niger* to synthesize a large panel of feruloyl and other cinnamoyl esterases when grown on agro-industrial by-products [33]. The strain *A. niger* BRFM281 has already been successfully used in a patented two-step process enabling the synthesis of vanillin from FA of maize bran. In the first step, *A. niger* released free FA from maize bran. In the second step, the bioconversion of free FA to vanillin was carried out by the food-grade basidiomycete *Pycnoporus cinnabarinus* [36]. In our case, a limiting step in the use of the fungus *A. niger*, when grown on RSM, was the degradation of the SA propenoic chain into syringic acid. The bottleneck was overcome by the use of the recombinant enzyme AnFaeA instead of the fungus. The recombinant *A. niger* AnFaeA used in this study was already characterized and produced, in our laboratory, at yields up to 1 g/L [28]. Feruloyl and cinnamoyl esterases (carboxyl ester hydrolases, EC 3.1.1), found especially in the genus *Aspergillus*, have been described as enzymes hydrolyzing ester linkages between hydroxycinnamic acid and sugars present in the plant cell wall [37]. In vivo, they have complex substrate specificities depending on the substituent present on the aromatic ring and the type of sugar bonding. For instance, type-A feruloyl esterases, such as AnFaeA, are active on the methyl esters of the highly substituted ferulic and sinapic acids, and on natural 1,5-esterified feruloylated oligomers from cereals, whereas type-B feruloyl esterases, such as AnFaeB, are active on the methyl esters of the hydroxylated cinnamic acids caffeic and *p*-coumaric acids, and on natural 1,2- and 1,6-esterified feruloylated oligomers from sugar beet pulp [38,39]. Pure AnFae were described as essentially inactive when used directly on the raw material [40]. In our case, we showed that (i) AnFaeA, AnFaeB and ChlE were induced in cultures of *A. niger* grown on RSM; and (ii) that pure AnFaeA could effectively hydrolyze sinapine (a choline ester of SA) from the meal. Moreover, the use of 39 nkat AnFaeA per gram RSM, at 55 °C and pH 5, permitted the total hydrolysis of sinapine and the recovery of free SA with a global yield of 68–76%.

*N. lepideus* is a basidiomycete of the *Polyporaceae* family and is currently one of the most consumed edible mushroom in Asia as well as the common mushroom *Agaricus bisporus* in Europe and North America. It was first described for its special ability to *O*-methylate or methoxylate *p*-cinnamic acids and to produce sweet flavors including *p*-methoxycinnamate, methyl cinnamate and methyl *p*-methoxycinnamate [41]. *N. lepideus* was also well-known as the agent responsible for the decay of railway sleepers and telegraph poles treated with creosote, a wood preservative, against which it showed an outstanding tolerance. More recently, *N. lepideus* was shown to produce biologically active compounds, e.g., the glycoprotein lepidane or soluble polysaccharides, with immunomodulating activities [42,43]. Extracts from carpophores of *N. lepideus* also possessed anti-radical and antioxidant activities [44]. Otherwise, *N. lepideus* demonstrated the particularity, rather exceptional in a basidiomycete, of assimilating and fermenting the pentose xylose from lignocellulosic biomasses to convert it into ethanol (a still limiting step in the synthesis of bioethanol 2G) with a yield of 0.3 g/g [45]. The species *N. lepideus* is thus of biotechnological interest. In our study, *N. lepideus* BRFM15 was the only fungus able to biotransform both free FA and SA to VG (a flavoring agent and a precursor of biopolymers of industrial interest) and canolol respectively, with concentrations up to 1.5 g/L. Interestingly, this suggested that *N. lepideus* did possess a PAD with very good affinity for both SA and FA. In addition, *N. lepideus* BRFM15 was also able to biotransform free *p*-coumaric acid and caffeic acid into 4-vinylphenol and 4-vinylcatechol respectively; however, it was unable to perform the bioconversion directly from raw natural materials (data not shown). GC–MS analysis of *N. lepideus* culture broth from SA to canolol bioconversion highlighted the presence of small traces of syringic acid, syringaldehyde and syringyl alcohol. In *N. lepideus* BRFM15 culture broth from FA to VG, were also detected as traces by GC–MS: vanillic acid, vanillin, vanillyl alcohol, syringic acid, syringaldehyde, syringyl alcohol, protocatechuic acid and derivatives of *p*-methoxycinnamate. In the light of the present work and reports on fungal aromatic metabolisms in the literature [46,47,48], it seemed that, for *N. lepideus*, the route of SA and FA biotransformation into the corresponding vinyl derivatives was highly favored compared to the others pathways. Especially, the bioconversion of hydroxycinnamic acids into the benzoic acid derivatives, via a *β*-oxidation-like reaction, is a minor pathway in *N. lepideus* BRFM15 unlike other *Polyporaceae* known for their aromatic flavor metabolism, including *P. cinnabarinus* [46] and *Trametes suaveolens* [47]. In our process, the concentrations of canolol obtained were up to 1.5 g/L. Among filamentous fungi, the synthesis of 0.75–1.15 mg/L canolol from 200 mg/L SA (potentially from olive mill wastewaters) has been recently described in *Phomopsis liquidambari*, a phytopathogenous ascomycete [48]. According to our batch assay, the global yield of the process was 3.3 mg canolol per gram of RSM, which was similar to the value obtained with the crude *Bacillus subtilis* PAD-process in water–toluene system [11]. In both cases, biotechnological treatments led to a 4.1 to 6.6-fold increase in canolol synthesis per gram of RSM, compared to heat treatments [20,21,22]. The use of specific adsorbents to trap aromatic compounds in culture broths has already been shown to be a safe and easy-to-handle method [49,50]. In our case, the same XAD2 resin could be used with high efficiency for both steps of the process, which constitutes an advantage for industrial scaling-up. To the best of our knowledge, this is the first report on a fungal/enzymatic bioconversion process of natural SA from RSM into canolol, compatible with sustainable biotechnological applications.

This work opens up new perspectives on the study and biotechnological use of fungal PADs. Indeed, these intracellular enzymes (generally homodimeric) responsible for the microbial non-oxidative decarboxylation of hydroxycinnamic acids have never been purified and characterized to our knowledge in filamentous fungi. Moreover, the substrate specificity of the native bacterial and yeast PADs was described mainly for ferulic, *p*-coumaric (pCA) and caffeic (CafA) acids, in the decreasing order [51,52]. Recently, a PAD from *Bacillus licheniformis* was shown to possess a broader substrate activity including pCA, FA, CafA, and SA decarboxylation but with the relative ratios of specific activities 100:75.6:34.4:0.3, respectively [53]. Obviously, cloning the *N. lepideus* PAD gene would enable to better understand the functioning, the specificity towards SA and the phylogeny of this enzyme.

## Figures and Tables

**Table 1:** Adsorption of SA and canolol for the different resins tested.

	Adsorption Ratio ^a^ (%)			
	pH 4		pH 7	
Type of adsorbent	SA	canolol	SA	canolol
XAD1180	94.2 ± 0.1	98.1 ± 0	37.7 ± 0.3	98.1 ± 0
XAD16	98.8 ± 0	99.1 ± 0	56.3 ± 1.0	99.2 ± 0
XAD2	84.9 ± 0.1	96.9 ± 0.2	30.8 ± 0.2	97.3 ± 0
SP207	99.3 ± 0	100 ± 0	75.2 ± 1.0	100 ± 0

^a^ Values are given as the mean ± standard deviation (*n* = 2).

## Supplementary Materials

Supplementary materials can be found at www.mdpi.com/2076-2607/5/4/67/s1.
