# Supplementary material for: A Two-Step Bioconversion Process for Canolol Production from Rapeseed Meal Combining an Aspergillus niger Feruloyl Esterase and the Fungus Neolentinus lepideus
Source: Microorganisms. 2017 Oct 14;5(4):67. doi: 10.3390/microorganisms5040067 (PMC5748576; doi:10.3390/microorganisms5040067)
Supplement: Supplementary file 1 [file microorganisms-05-00067-s001.pdf]

Table S1. Sinapic acid molar mass balance after AnFaeA hydrolysis of RSM (55°C, 39 nkat AnFaeA per gram of RSM).

	Sinapine (μmol/g DM)[a]	SA (μmol/g DM)[a]	Total SA (the free and the choline ester forms) (μmol SA/g DM)[a]
Initial raw RSM composition	25.8 ± 1.7	2.2 ± 0.04	28
Reaction mixture composition after 0.5 h incubation of RSM in buffer[b]	14.5 ± 0.3	1.78 ± 0	16.28
Reaction mixture composition after 3.5 h incubation of RSM in buffer[b] in the presence of AnFaeA	0	29.88 ± 0.4	29.88

[a] Values are given as the mean ± standard deviation (n=2)

[b] 100 mM MOPS buffer (pH 5)

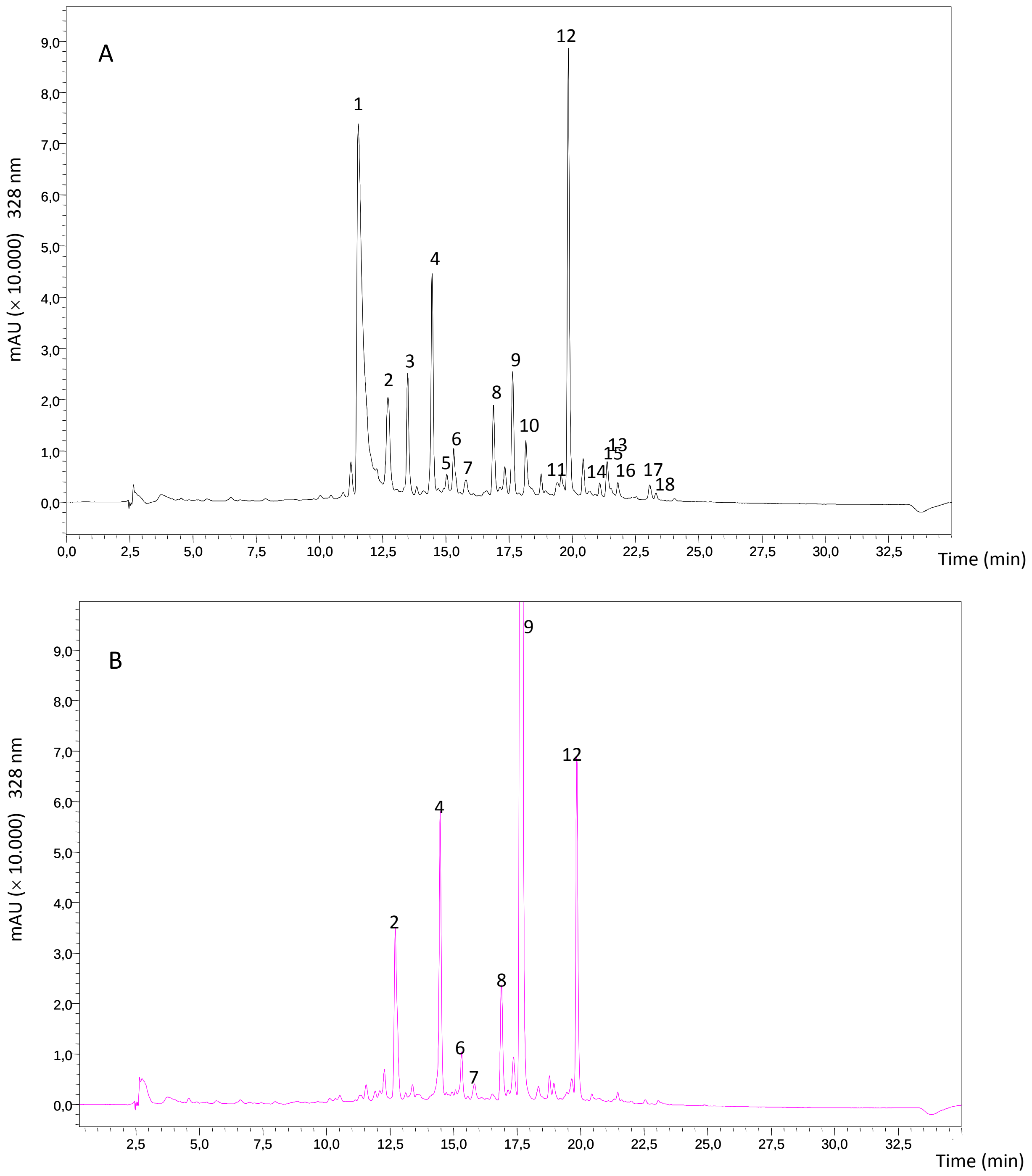

Figure S1. HPLC chromatograms of: (A) Reaction mixture after 0.5 h incubation of RSM in 100 mM MOPS buffer (pH 5) at 55°C; (B) Reaction mixture after 3.5 h incubation of RSM in 100 mM MOPS buffer (pH 5) at 55°C in the presence of 39 nkat AnFaeA per gram of RSM.

Peaks: 1: sinapine; 2: kaempherol sophoroside; 3: glucopyranosyl sinapate; 4: kaempherol sinapoyl trihexoside; 5-8: not determined; 9: trans-sinapic acid; 10: cis-sinapic acid; 11: kaempherol sinapoyl trihexoside; 12 and 13: disinapoyl gentiobioside; 14: not determined; 15: disinapoyl β-glucopyranoside; 16: not determined; 17 and 18: trisinapoyl gentiobioside.

```
Seq 1 Schizophyllum       MPGTWEEDLKDVHLLYDYDVKQPDGSTEKWRYELWCSHENRVTYAIHGGPMAGRYNYQET
Seq 2 Stereum             -MDHFDKDIRDVHLLYDYDVMGEGGNPEKWRYEMWFFSEKRIVYSIHGGPMAGRLNYQTC
Seq 3 Neolentinus         -MSHEGATSEEFKQIEGKRFKYTYG--LGWTYEMYFRSLTRCVYRILSGPLAGRVNFQHA
                            .      .:.: : .  .    *    * **::    .* .* * .**:*** *:*

Seq 1 Schizophyllum       KYQCIRPGELWQINWLEETGTIVSIVYDILKQRITTLIAFSKGHWEHSVEAHGDKRNPAD
Seq 2 Stereum             EFQCIRPGELWQCNWLEETGTIVSLVYDIPRKRITTMIGFSKGHWEHAKEAHGDKRNPAD
Seq 3 Neolentinus         HYQKIRDN-VWQCSWLEETGTVVSMVVDFDQQTVKTFATFSRGHWDLPDQAKGWKRNPED
                          .:* ** . :** .*******:**:* *: :: :.*:  **:***: . :*:* **** *

Seq 1 Schizophyllum       LERWRGLAKIG-TQTDRYLLAEQADIVKNFKGPGDLKPIDLSWPTL
Seq 2 Stereum             FERWRALAKIG-TQTDRHILCEQADILEVFKGKGDLVPIEPDAETL
Seq 3 Neolentinus         MARWRTLAQKGKDQADKHVLVEHAKMSELTSGQGDLPDIDDSWETM
                          : *** **: *  *:*:::* *:*.: :  .* ***  *: .  *:
```

Figure S2. ClustalW alignment of the protein sequences of the fungal PADs predicted from the genome of the strains *Neolentinus lepideus* HHB14362, *Schizophyllum commune* H4-8 and *Stereum hirsutum* FP-91666 (Accession Numbers in the NCBI database: KZT30061.1, XP_003032860.1, and XP_007303961.1, respectively [31]).

Sequences 3 and 1, and sequences 3 and 2 showed 36.4 and 38.3% similarity, respectively.

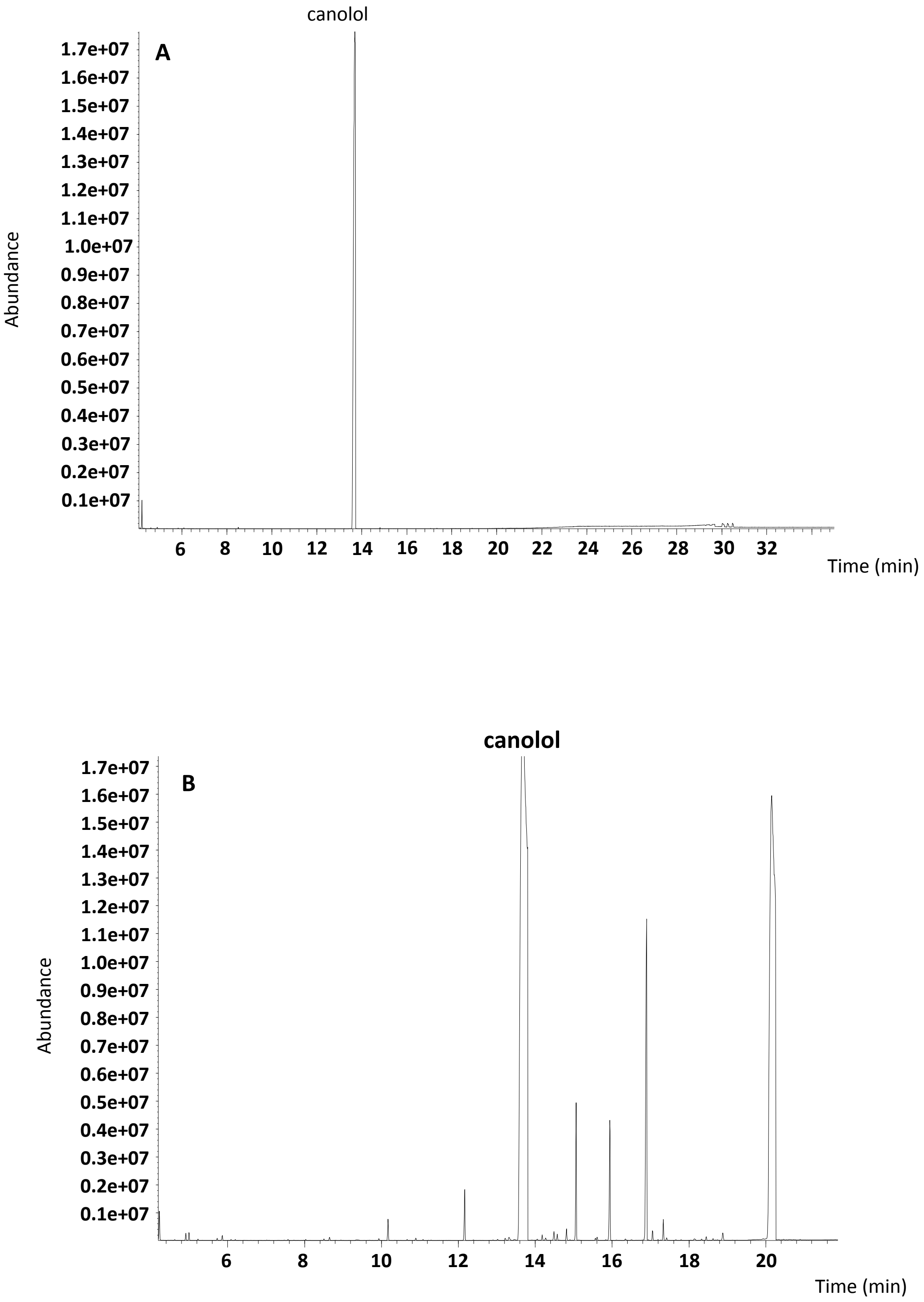

Figure S3. GC chromatograms of: (A) the silylated derivative of the canolol standard; (B) the silylated derivatives of the phenolics from the *N. lepideus* BRFM15 culture broth supplemented with sinapic acid as the substrate of bioconversion.

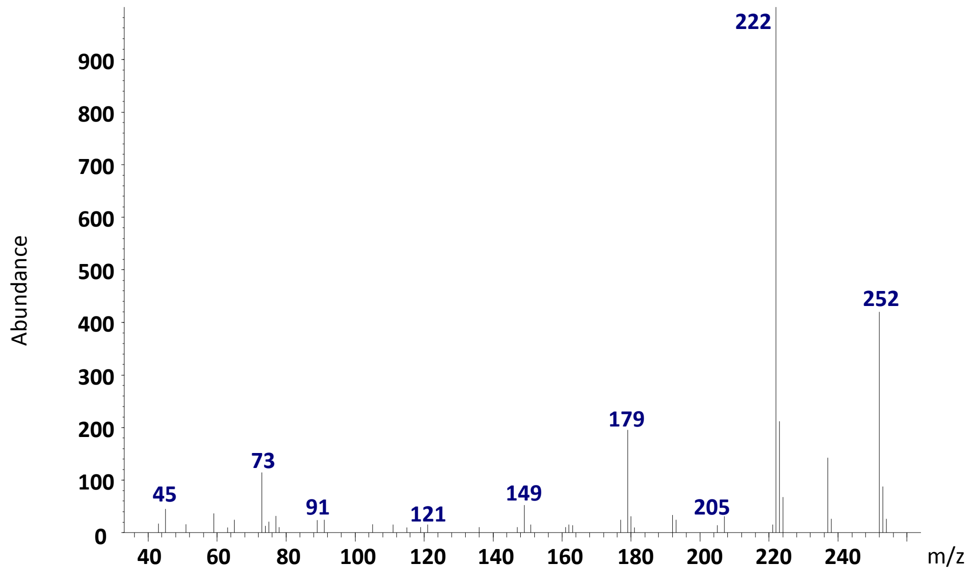

Figure S4. MS fragmentation of canolol (silylated derivative) with the following ions: **252 (42)**, 237 (14), 222 (100), 207 (3), 205 (1), 192 (3), 179 (19), 149 (5), 73 (5). In bold, the molecular ion; in parenthesis, the ion relative intensity.

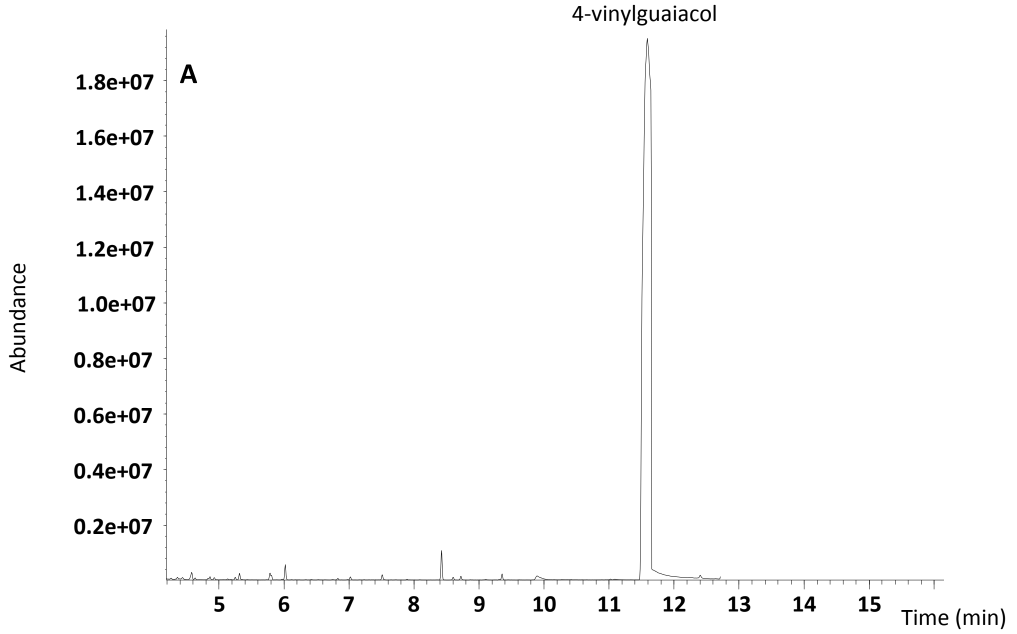

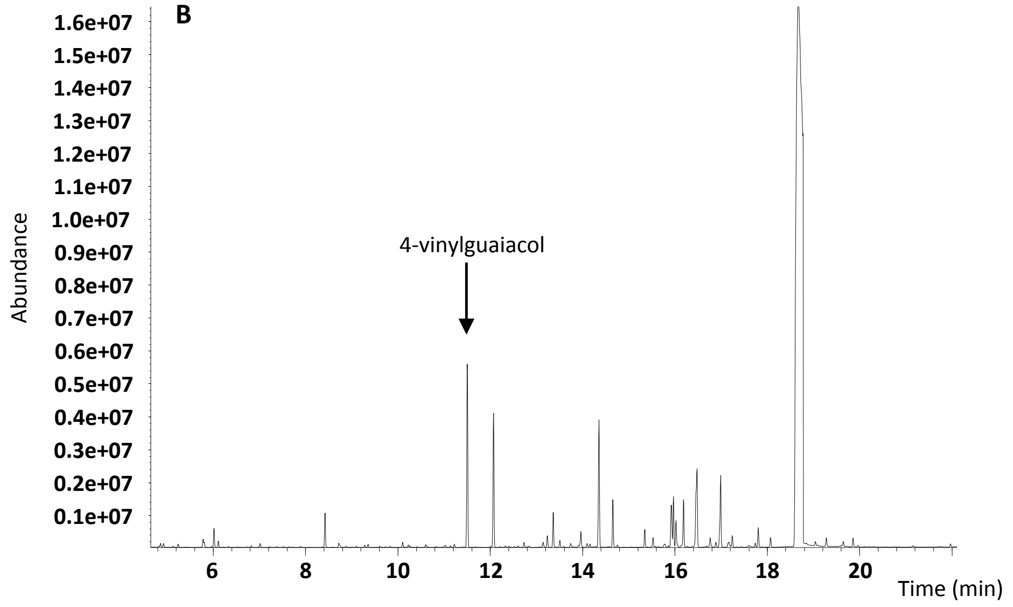

Figure S5. GC chromatograms of: (A) the silylated derivative of the 4-vinylguaicol standard; (B) the silylated derivatives of the phenolics from the *N. lepideus* BRFM15 culture broth supplemented with ferulic acid as the substrate of bioconversion.

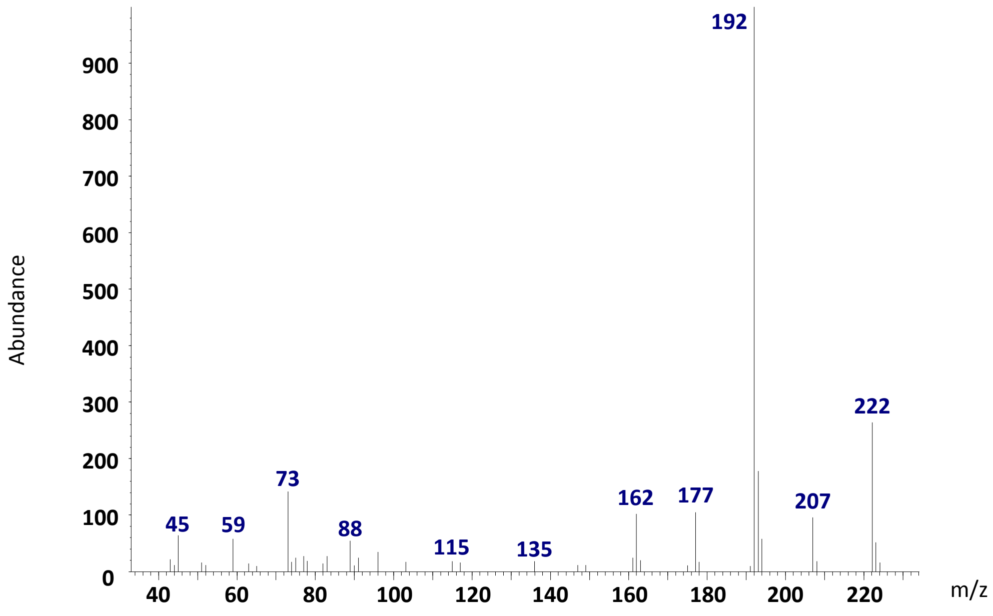

Figure S6. MS fragmentation of 4-vinylguaiacol (silylated derivative) with the following ions: **222 (31)**, 207 (11), 192 (100), 193 (18), 177 (10), 162 (10), 136 (2), 73 (10). In bold, the molecular ion; in parenthesis, the ion relative intensity.
